# The prognosis and management of reclassified systemic lupus erythematosus associated pulmonary arterial hypertension according to 2022 ESC/ERS guidelines

**DOI:** 10.1186/s13075-024-03338-1

**Published:** 2024-05-27

**Authors:** Yutong Li, Junyan Qian, Xingbei Dong, Jiuliang Zhao, Qian Wang, Yanhong Wang, Xiaofeng Zeng, Zhuang Tian, Mengtao Li

**Affiliations:** 1 Department of Rheumatology and Clinical Immunology, Chinese Academy of Medical Sciences & Peking Union Medical College, National Clinical Research Center for Dermatologic and Immunologic Diseases (NCRC-DID), Ministry of Science & Technology, State Key Laboratory of Complex Severe and Rare Diseases Key Laboratory of Rheumatology and Clinical Immunology, Ministry of Education, Peking Union Medical College Hospital, Peking Union Medical College Hospital (PUMCH), Beijing, 100730 China; 2 Department of Cardiology, Dongcheng District, Peking Union Medical College Hospital, No.1 Shuai Fu Yuan, Wang Fu Jing, Beijing, 100730 China; 3 Department of Epidemiology and Bio-Statistics, Institute of Basic Medical Sciences, China Academy of Medical Sciences & Peking Union Medical College, Beijing, China

**Keywords:** System lupus erythematosus associated pulmonary arterial hypertension, Hemodynamics

## Abstract

**Background and aims:**

The 2022 European Society of Cardiology/European Respiratory Society (ESC/ERS) guideline has recently revised the hemodynamic definition of pulmonary arterial hypertension. However, there is currently limited research on the prognosis and treatment of system lupus erythematosus-associated pulmonary arterial hypertension (SLE-PAH) patients that have been reclassified by the new hemodynamic definition. This study aims to analyze the prognosis of newly reclassified SLE-PAH patients and provide recommendations for the management strategy.

**Methods:**

This retrospective study analyzed records of 236 SLE-PAH patients who visited Peking Union Medical College Hospital (PUMCH) from 2011 to 2023, among whom 22 patients were reclassified into mild SLE-PAH (mean pulmonary arterial pressure (mPAP) of 21–24 mmHg, pulmonary vascular resistance (PVR) of 2–3 WU, and PAWP ≤ 15 mmHg) according to the guidelines and 14 were defined as unclassified SLE-PAH patients (mPAP 21–24 mmHg and PVR ≤ 2 WU). The prognosis was compared among mild SLE-PAH, unclassified SLE-PH, and conventional SLE-PAH patients (mPAP ≥ 25 mmHg and PVR > 3WU). Besides, the effectiveness of pulmonary arterial hypertension (PAH)-specific therapy was evaluated in mild SLE-PAH patients.

**Results:**

Those mild SLE-PAH patients had significantly longer progression-free time than the conventional SLE-PAH patients. Among the mild SLE-PAH patients, 4 did not receive PAH-specific therapy and had a similar prognosis as patients not receiving specific therapy.

**Conclusions:**

This study supports the revised hemodynamic definition of SLE-PAH in the 2022 ESC/ERS guideline. Those mild and unclassified SLE-PH patients had a better prognosis, demonstrating the possibility and significance of early diagnosis and intervention for SLE-PAH. This study also proposed a hypothesis that IIT against SLE might be sufficient for those reclassified SLE-PAH patients.

**Supplementary Information:**

The online version contains supplementary material available at 10.1186/s13075-024-03338-1.

## Introduction

PAH is one of the most severe complications of system lupus erythematosus (SLE). pulmonary arterial hypertension (PAH) has also been demonstrated to be an important death cause in SLE patients [1, 2]. Epidemiological research has revealed that SLE is the first predominant cause of connective tissue disease (CTD)-associated PAH in Asian nations [3–7].

The previous definition of pulmonary hypertension (PAH) was described as mPAP ≥ 25 mmHg, PVR > 3WU and pulmonary arterial wedge pressure (PAWP) ≤ 15 mmHg [8]. However, research has proven that mildly elevated mPAP and PVR lead to elevated mortality and a worse prognosis [9–15]. Therefore, the 2022 ESC/ERS guideline updated the definition of PAH to mPAP > 20 mmHg, PVR > 2WU, and PAWP ≤ 15 mmHg [16]. The change in diagnostic criteria redefined those patients with mild PAH and contributed to their early diagnosis and detection. SLE-PAH patients dominate the Asian CTD-PAH patients and have a mortality rate exceeding 14% [17]. Early diagnosis and intervention are expected to improve their prognosis and prevent further disease progression. However, there is currently limited study focus on SLE-PAH, which demonstrates how the revised hemodynamic definitions impact the prognosis of SLE-PAH. Furthermore, only patients who meet the conventional hemodynamic criteria have demonstrated the efficacy of PAH-specific drugs against PAH, and the lack of available data to guide the management of these reclassified SLE-PAH patients hinders the application of the new guideline in clinical practice [16].

The aims of this study are to (i) analyze the prognosis of those reclassified SLE-PAH patients and prove the impact of early diagnosis and detection; (ii) evaluate the effectiveness of PAH-specific therapy in these patients and provide recommendations for their management.

## Methods

### Patients

**Fig. 1:**
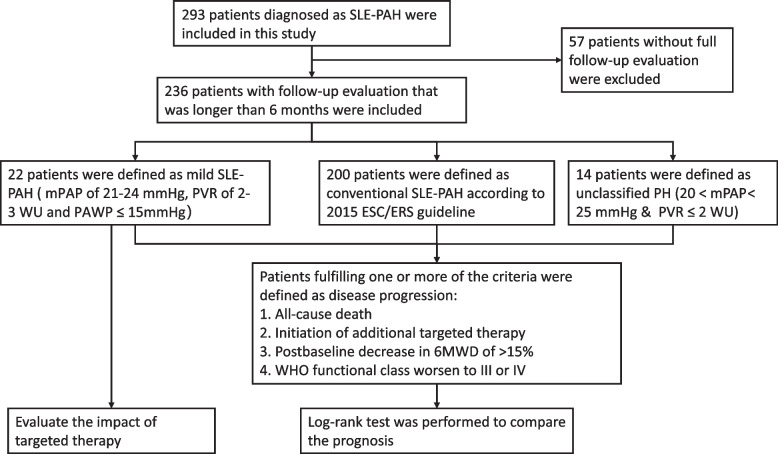
Flow chart of the study design

The Chinese SLE Treatment and Research Group-PAH (CSTAR-PAH) is the largest national cohort that follows up on SLE-PAH patients in China [18]. This study is a retrospective study based on the CSTAR-PAH cohort. All of the enrolled patients visited the Peking Union Medical College Hospital (PUMCH) and fulfilled the 2012 Systemic Lupus International Collaborating Clinics (SLICC) classification criteria for SLE [19, 20]. Besides, all the participants were diagnosed with PAH via right heart catheterization (RHC) or transthoracic echocardiography (TTE). All the patients underwent their first RHC test between 2011 to 2023. Patients fulfilled the following criteria: 1) mPAP ≥ 25 mmHg; 2) PVR > 3 WU; 3) pulmonary arterial wedge pressure (PAWP) ≤ 15 mmHg were defined as conventional SLE-PAH. Other patients were reclassified as mild SLE-PAH if they met the hemodynamic criteria of mPAP of 21–24 mmHg, PVR of 2–3 WU, and PAWP ≤ 15 mmHg. Patients with mPAP of 21–24 mmHg and PVR ≤ 2 were reclassified as unclassified SLE-PH. The unclassified SLE-PH patients had all been diagnosed as SLE-PAH via TTE but had not undergone RHC previously. The special hemodynamic parameters of unclassified SLE-PH patients attributed to previous PAH-specific therapy. Patients lost follow-up or had insufficient follow-up time were excluded. Patients with other connective tissue diseases (CTD) such as systemic sclerosis (SSc) were excluded. Patients with other types of pulmonary hypertension (PH) revealed by PAWP > 15mmg, diffusing capacity of the lung for carbon monoxide (DLCO) < 60% or pulmonary embolism diagnosed by ventilation perfusion scintigraphy or computed tomographic pulmonary angiography (CTPA). Patients with a follow-up time shorter than 6 months were also excluded. The flow chart of the research process is shown in Fig. 1. The Peking Union Medical College Hospital Institutional Review Board and Ethical Board approved this study (ethic number JS-2038). All patients gave written informed permission.

The process of this study was demonstrated in the Figure. 293 patients diagnosed as SLE-PAH were included in this study and classified to three groups according to their RHC result. Prognosis of three groups were compared and the effect of PAH-specific therapy was evaluated.

### Data collection and risk assessment

The CSTAR cohort is not an inception cohort since some patients were diagnosed with PAH in other hospitals via transthoracic echocardiography (TTE) and treated. The baseline was defined as the first RHC the patients underwent. The demographic features, clinical evaluation, laboratory results, medical treatment, TTE, and RHC parameters were recorded at baseline. The minimum follow-up time for the patients was 6 months. IIT was defined as using one or more of cyclophosphamide (CTX), mycophenolate mofetil (MMF), cyclosporin A (CsA), azathioprine (AZA), and tacrolimus (FK506).

The progression of PAH was defined as (i) all cause death; or (ii) hospitalization for more than 24 h due to deterioration of PAH; or (iii) additional PAH-specific therapy compared to baseline; or (iv) a decrease in 6-min walk distance (6MWD) of more than 15%; or (v) deterioration of the World Health Organization functional class (WHO-FC) to level III or IV [21].

The COMPERA 2.0 four-strata risk assessment tool and three-strata model published in the 2022 ESC/ERS guideline were applied to evaluate the baseline condition of SLE-PAH patients [16, 22].

### Statistical analysis

Quantitative variables were described as means and standard deviations. Non-quantitative data were presented as counts and percentages. *Tableone* R package (R 4.3.1) was used to compare the baseline characteristics between different groups. Kaplan–Meier estimation was used to calculate cumulative survival probabilities, and log-rank test was used to compare the survival rates between different groups (conventional SLE-PAH, mild SLE-PAH, and unclassified SLE-PH). Log-rank test was also performed to compare the prognosis of mild SLE-PAH patients with/without PAH-specific therapy. Elevation of risk strata of one or more variables or additional utilization of PAH-specific drugs compared to baseline was defined as endpoint events as mentioned above. The survival time was calculated from the baseline time. The *survival* and *survminer* R packages were used to perform survival analysis (R 4.3.1). The values of *p* < 0.05 were considered statistically significant.

## Results

### Study population and baseline characteristics

In this study, a total of 236 SLE-PAH patients were finally included, among whom 36 met the reclassifying criteria as previously stated. 22 reclassified patients were defined as mild SLE-PAH, and 14 were defined as unclassified SLE-PH. Baseline characteristics of patients are displayed in Table 1. There was no significant disparity in the demographic characteristics between the three groups with different hemodynamic profiles. The majority of enrolled patients were female (97.5%) and the mean age when they were recruited was around 35. The disease activity and manifestation of SLE also showed no statistically significant difference. The mean SLE disease activity index (SLEDAI) also showed similarity between different groups [23]. The three groups showed significant differences in PAH risk assessment indicators. There were only 14.3% of the mild patients in WHO FC (World Health Organization functional class) III, while 27% of conventional patients were in WHO FC III or IV. The reclassified SLE-PAH patients also had a higher mean 6MWD (511.6 m for mild patients and 586 m for unclassified SLE-PH patients) than the conventional ones (496.6 m). Meanwhile, the overall risk strata of mild SLE-PAH patients and unclassified SLE-PH patients were significantly lower than those of conventional patients.

**Table 1:**
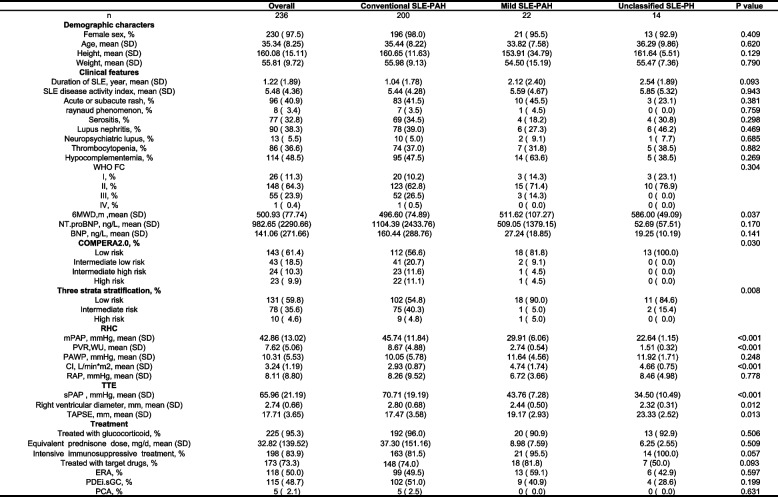
Demographic and clinical data at baseline

### Prognosis of reclassified SLE-PAH patients

**Fig. 2:**
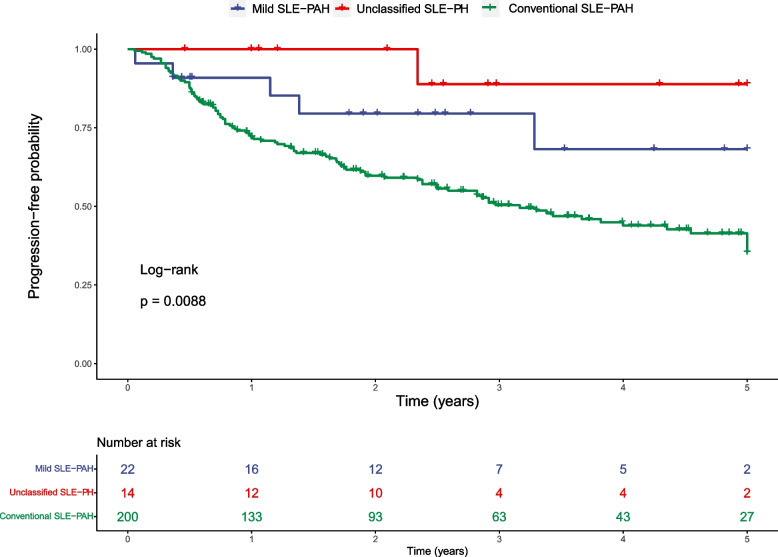
Comparison of prognosis between three groups

To assess the prognosis of SLE-PAH patients, we defined the endpoint event as disease progression. A detailed definition of disease progression is shown above. Overall, 5 mild SLE-PAH, 1 unclassified SLE-PH and 102 conventional SLE-PAH patients progressed during the follow-up. The log-rank test demonstrates that the mild SLE-PAH group and the unclassified SLE-PH group had significantly better prognosis than the conventional SLE-PAH group (Fig. 2), while the two groups of reclassified SLE-PAH showed similar prognosis (Figure S1).

The prognosis was evaluated by disease progression rate of conventional SLE-PAH (green), mild SLE-PAH (blue) and unclassified SLE-PH (red) groups. The log-rank test p value was shown in the figure.

### Efficacy of PAH-specific therapy for mild SLE-PAH patients

The 22 mild SLE-PAH patients are extracted for further analysis to study the impact of PAH-specific therapy on their prognosis. The comparision of baseline characteristics for 22 mild SLE-PAH patients, who were grouped according to the utilization of PAH-specific therapy, is shown in Table 2. The baseline characteristics of patients with or without PAH-specific therapy showed no significant disparity. Among the mild SLE-PAH patients, 4 patients did not receive PAH-specific therapy at baseline, and none of them progressed during the follow-up. As shown in Table S1, there was no significant difference between the prognosis of patients with or without PAH-specific therapy. All five progressed patients were defined as progressing due to receiving additional PAH-specific therapy to prevent further deterioration. It is worth noting that almost all reclassified patients, no matter whether they had mild SLE-PAH or unclassified SLE-PH, received IIT.

**Table 2:**
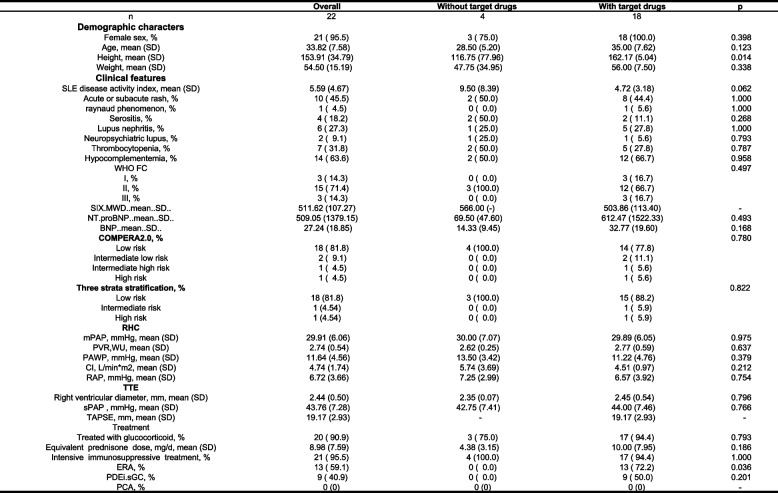
Demographic and clinical data of mild SLE-PAH patients at baseline

## Discussion

To our knowledge, this is the first study focusing on the SLE patients that were newly classified as SLE-PAH according to the 2022 ESC/ERS guideline [16]. In the CSTAR cohort, 22 patients (15.3%) from the PUMCH center were reclassified to mild SLE-PAH and 14 to unclassified SLE-PH according to the new hemodynamic definition proposed in the 2022 ESC/ERS guideline. Their prognosis was compared with that of conventional SLE-PAH patients [8]. Considering the current lack of evidence-based data to guide the treatment of such mild SLE-PAH patients, we analyze the impact of PAH-specific therapy on the prognosis.

### Reclassified SLE-PAH patients had better prognosis

The conventional diagnosis criteria for PAH were set as mPAP ≥ 25 mmHg, PVR > 3 WU and PAWP ≤ 15 mmHg. However, previous research demonstrated that mildly elevated mPAP and PVR could cause elevated mortality and a worse prognosis [9–15]. Therefore, the 2022 ESC/ERS guidelines revised the hemodynamic definition of PAH, which reclassified some patients into PAH [16] The new diagnostic criteria emphasized the diagnosis of patients at the early stage of the disease. As for CTD-PAH, previous research mainly focused on systemic sclerosis associated PAH (SSc-PAH), since SSc-PAH dominates the patient population in Europe and America [24–26]. The efficacy of early detection of SSc-PAH with the DETECT algorithm according to new definition has been validated. The sensitivity of the algorithm reached 88.2% and contributed to the early diagnosis of SSc-PAH [27]. Recent research on SSc-PAH demonstrated that 53.3% of the patients who had not been diagnosed of PH were reclassified to pre-capillary PH according to the 2022 ESC/ERS guidelines, and those patients had a better prognosis [28]. In this study, we focused on SLE patients who did not meet the previous PAH diagnosis criteria but were reclassified to PAH or unclassified PH according to the 2022 ESC/ERS guidelines. We grouped those reclassified patients into mild SLE-PAH and unclassified PH group according to their mPAP and PVR, and compared the prognosis between the groups. The risk of progression for mild SLE-PAH and unclassified SLE-PH patients was proven to be lower than those convention SLE-PAH patients. It indicated that early PAH-specific treatment in SLE-PAH patients with relatively good hemodynamic conditions can help improve their prognosis. A prediction model for the risk of PAH in SLE patients were developed, and enetic risk factors for SLE-PAH such as HLA-DQA1*03:02 have been discovered [29, 30]. With further advancement in the research of molecular mechanisms and genetic markers of SLE-PAH, it is expectable that more accurate and comprehensive prediction models, such as the polygenic risk score (PRS), will be constructed in the future, which will enable the early diagnosis and treatment of SLE-PAH, thereby improving patient prognosis.

### Treatment strategy of mild SLE-PAH patients

Treatment of CTD-PAH can be divided into specific drugs against PAH and immunosuppressive therapy against CTD. The PAH-specific therapy for IPAH was recommended to treat CTD-PAH adhering to nearly the same algorithm [16]. When it comes to the other part of the treatment, rituximab has been proven to be an efficient and safe drug to treat SSc-PAH by depleting B cells [31], which strongly supports the opinion that SSc-PAH patients can benefit from immunosuppressive therapy. Cyclophosphamide, in combination with glucocorticoids, was reported to be beneficial for the PAH of SLE-PAH patients [32]. Besides, IIT in combination with PAH-specific therapy for PAH was proven to improve the prognosis of SLE-PAH patients [33]. The efficacy of immunosuppressive therapy was attributed to the significant role played by the inflammatory response of CTD in the pathogenesis of CTD-PAH, which has been demonstrated in both patients and animal models [34, 35]. The study of treatment in SLE involving other organs, such as immunosuppressive therapy for lupus nephritis, could also provide some clues [36, 37]. With these evidences, our team proposed the concept of “dual treat-to-target” for SLE-PAH and other type of CTD-PAH patients, attaching importance to CTD related immunosuppressive therapy and PAH related PAH-specific therapy [38]. In this study, 22 mild SLE-PAH patients with follow-up time longer than 6 months are grouped based on whether they received PAH-specific therapy. We compared the prognosis between the two groups, and the result shows that the two groups had a similar prognosis. Besides, the unclassified SLE-PH patients had similar prognosis compared to those mild patients, while only half of them received PAH-specific therapy. Though the statistical test was limited by the sample size of this study, the results could also give us some clues. Therefore, we proposed a hypothesis that IIT might be sufficient for SLE-PAH patients with a mild change in hemodynamic parameters.

### Strengths and limitations

This is the first study that focuses on the prognosis of reclassified SLE-PAH patients according to the new hemodynamic definition based on the CSTAR-PAH cohort in China [16]. This study also proposes a hypothesis that IIT might be sufficient for mild SLE-PAH patients.

However, this study has several limitations. First, this is not a randomized clinical trial to evaluate the efficacy of PAH-specific therapy, the detailed treatment plans and baseline condition of different patients varied. Further standardized study is needed. Second, though our cohort is the currently the largest SLE-PAH cohorts in China, the sample size is still small, which restricts us from conducting more detailed studies. Further study with a larger sample size is called in the future.

## Conclusion

This study supports the revised hemodynamic definition of SLE-PAH in the 2022 ESC/ERS guideline. Those reclassified SLE-PAH patients had a better prognosis compared to conventional SLE-PAH patients. It demonstrates the significance of early diagnosis and intervention for SLE-PAH.

### Supplementary Information

Supplementary Material 1.

Supplementary Material 2.

## Data Availability

No datasets were generated or analysed during the current study.

## Abbreviations

ACR: American College of Rheumatology
AZA: Azathioprine
CsA: Cyclosporin A
CSTAR-PAH: Chinese SLE Treatment and Research Group-Pulmonary Arterial Hypertension
CTD: Connective tissue disease
CTD-PAH: Connective tissue disease associated pulmonary arterial hypertension
CTPA: Computed tomographic pulmonary angiography
CTX: Cyclophosphamide
DLCO: Diffusing capacity of the lung for carbon monoxide
FK506: Tacrolimus
IIT: Intensive immunosuppressive therapy
IPAH: Idiopathic pulmonary arterial hypertension
MMF: Mycophenolate mofetil
PAH: Pulmonary arterial hypertension
PAWP: Pulmonary arterial wedge pressure
PH: Pulmonary hypertension
PRS: Polygenic risk score
PUMCH: Peking Union Medical College Hospital
PVR: Pulmonary vascular resistance
RCT: Randomized controlled trials
RHC: Right heart catheterization
SLEDAI: System lupus erythematosus disease activity index
SLE-PAH: System lupus erythematosus associated pulmonary arterial hypertension
SLICC: Systemic Lupus International Collaborating Clinics
SSc: Systemic sclerosis
SSc-PAH: Systemic sclerosis associated pulmonary arterial hypertension
TTE: Transthoracic echocardiography
WHO-FC: World Health Organization functional class
6MWD: 6-Minute walk distance
