# Supplementary figures and images for: The prognosis and management of reclassified systemic lupus erythematosus associated pulmonary arterial hypertension according to 2022 ESC/ERS guidelines
Source: Arthritis Res Ther. 2024 May 27;26:109. doi: 10.1186/s13075-024-03338-1 (PMC11129383; doi:10.1186/s13075-024-03338-1)

Progression-free probability

Mild SLE-PAH    Unclassified SLE-PH

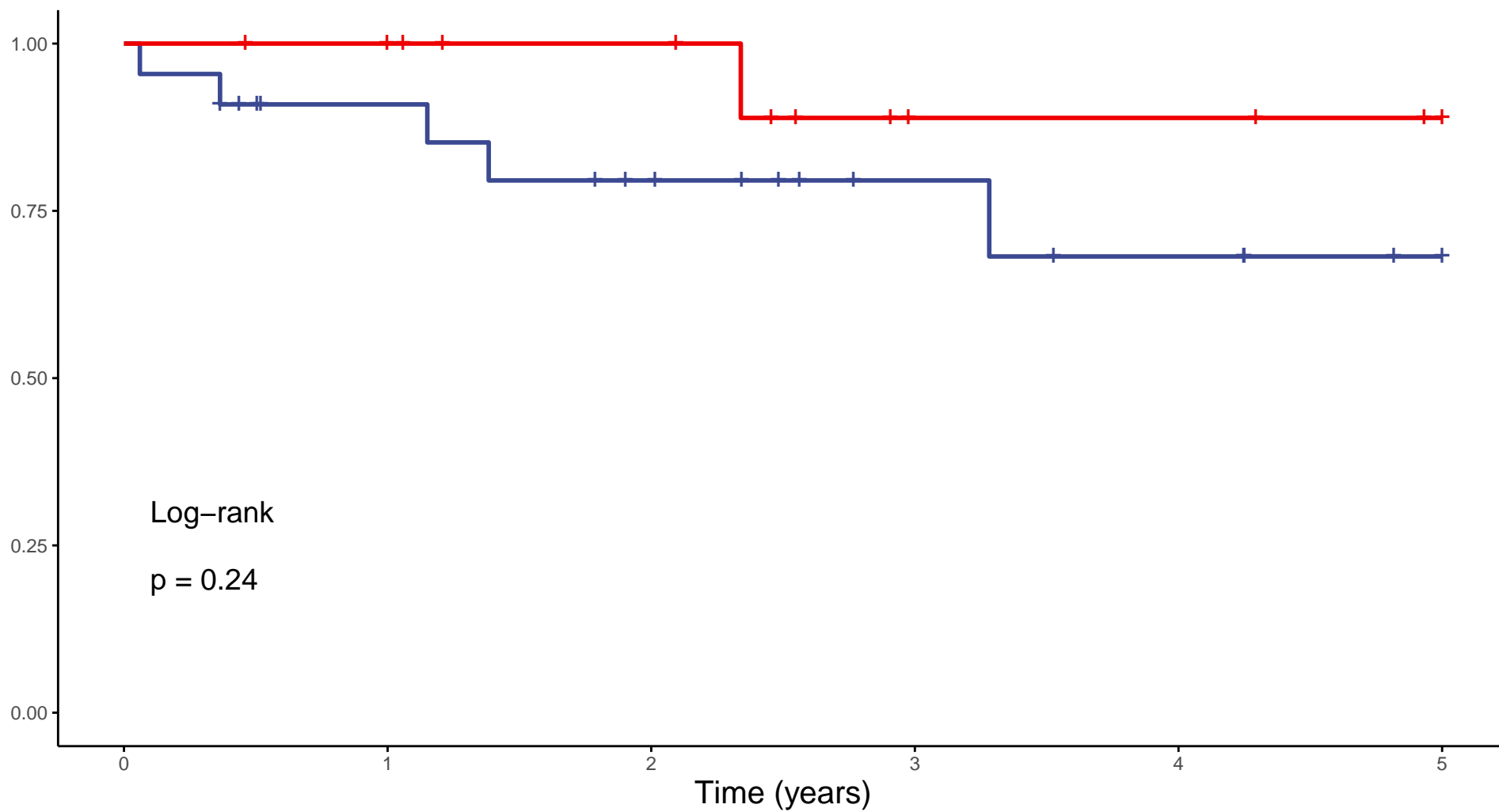

Number at risk

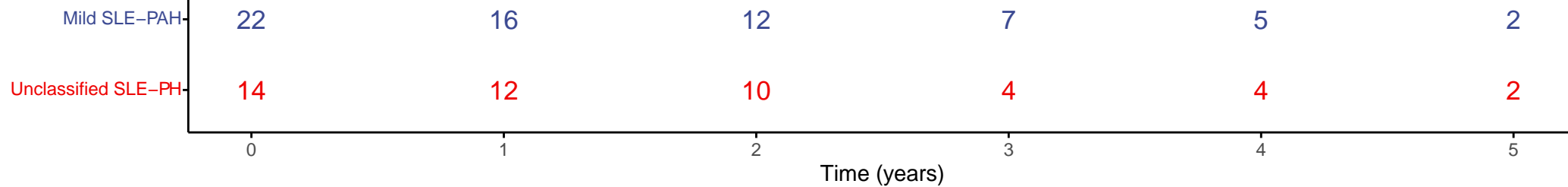

Supplement: Supplementary file 1 — Supplementary Material 1. [file 13075_2024_3338_MOESM1_ESM.pdf]
