# Supplementary material for: The prognosis and management of reclassified systemic lupus erythematosus associated pulmonary arterial hypertension according to 2022 ESC/ERS guidelines
Source: Arthritis Res Ther. 2024 May 27;26:109. doi: 10.1186/s13075-024-03338-1 (PMC11129383; doi:10.1186/s13075-024-03338-1)
Supplement: Supplementary file 2 — Supplementary Material 2. [file 13075_2024_3338_MOESM2_ESM.pdf]

Table S1. Demographic and clinical data of mild SLE-PAH patients at baseline

	**Overall**	**Without target drugs**	**With target drugs**	**p**
n	22	4	18	
**Demographic characters**				
Female sex, %	21 (95.5)	3 (75.0)	18 (100.0)	0.398
Age, mean (SD)	33.82 (7.58)	28.50 (5.20)	35.00 (7.62)	0.123
Height, mean (SD)	153.91 (34.79)	116.75 (77.96)	162.17 (5.04)	0.014
Weight, mean (SD)	54.50 (15.19)	47.75 (34.95)	56.00 (7.50)	0.338
**Clinical features**				
SLE disease activity index, mean (SD)	5.59 (4.67)	9.50 (8.39)	4.72 (3.18)	0.062
Acute or subacute rash, %	10 (45.5)	2 (50.0)	8 (44.4)	1.000
raynaud phenomenon, %	1 (4.5)	0 (0.0)	1 (5.6)	1.000
Serositis, %	4 (18.2)	2 (50.0)	2 (11.1)	0.268
Lupus nephritis, %	6 (27.3)	1 (25.0)	5 (27.8)	1.000
Neuropsychiatric lupus, %	2 (9.1)	1 (25.0)	1 (5.6)	0.793
Thrombocytopenia, %	7 (31.8)	2 (50.0)	5 (27.8)	0.787
Hypocomplementemia, %	14 (63.6)	2 (50.0)	12 (66.7)	0.958
WHO FC				0.497
I, %	3 (14.3)	0 (0.0)	3 (16.7)	
II, %	15 (71.4)	3 (100.0)	12 (66.7)	
III, %	3 (14.3)	0 (0.0)	3 (16.7)	
SIX.MWD..mean..SD..	511.62 (107.27)	566.00 (-)	503.86 (113.40)	-
NT.proBNP..mean..SD..	509.05 (1379.15)	69.50 (47.60)	612.47 (1522.33)	0.493
BNP..mean..SD..	27.24 (18.85)	14.33 (9.45)	32.77 (19.60)	0.168
**COMPERA2.0, %**				0.780
Low risk	18 (81.8)	4 (100.0)	14 (77.8)	
Intermediate low risk	2 (9.1)	0 (0.0)	2 (11.1)	
Intermediate high risk	1 (4.5)	0 (0.0)	1 (5.6)	
High risk	1 (4.5)	0 (0.0)	1 (5.6)	
**Three strata stratification, %**				0.822
Low risk	18 (81.8)	3 (100.0)	15 (88.2)	
Intermediate risk	1 (4.54)	0 (0.0)	1 (5.9)	
High risk	1 (4.54)	0 (0.0)	1 (5.9)	
**RHC**				
mPAP, mmHg, mean (SD)	29.91 (6.06)	30.00 (7.07)	29.89 (6.05)	0.975
PVR,WU, mean (SD)	2.74 (0.54)	2.62 (0.25)	2.77 (0.59)	0.637
PAWP, mmHg, mean (SD)	11.64 (4.56)	13.50 (3.42)	11.22 (4.76)	0.379
CI, L/min*m2, mean (SD)	4.74 (1.74)	5.74 (3.69)	4.51 (0.97)	0.212
RAP, mmHg, mean (SD)	6.72 (3.66)	7.25 (2.99)	6.57 (3.92)	0.754
**TTE**				
Right ventricular diameter, mm, mean (SD)	2.44 (0.50)	2.35 (0.07)	2.45 (0.54)	0.796
sPAP , mmHg, mean (SD)	43.76 (7.28)	42.75 (7.41)	44.00 (7.46)	0.766
TAPSE, mm, mean (SD)	19.17 (2.93)	-	19.17 (2.93)	-
Treatment				
Treated with glucocorticoid, %	20 (90.9)	3 (75.0)	17 (94.4)	0.793
Equivalent prednisone dose, mg/d, mean (SD)	8.98 (7.59)	4.38 (3.15)	10.00 (7.95)	0.186
Intensive immunosuppressive treatment, %	21 (95.5)	4 (100.0)	17 (94.4)	1.000
ERA, %	13 (59.1)	0 (0.0)	13 (72.2)	0.036
PDEi.sGC, %	9 (40.9)	0 (0.0)	9 (50.0)	0.201
PCA, %	0 (0)	0 (0)	0 (0)	-

Table S2. Impact of treatment with target drug on prognosis of mild SLE-PAH patients

	**Without targeted drugs**	**With targeted drugs**
Number of patients	18	4
Number of progressed patients	5	0
Survival time of progressed patients (years)	0.06, 0.36,1.15, 1.38, 3.28	-
Log-rank test p value	0.25	

Table S3. SLICC/ACR Damage index of patients at baseline

	Overall	Conventional SLE-PAH	Mild SLE-PAH	Unclassified SLE-PH	P value
n	236	200	22	14	
OCULAR					
Any cataract ever, %	2 (0.8)	0 (0.0)	0 (0.0)	2 (1.0)	0.834
Retinal change OR Optic atrophy, %	0 (0)	0 (0)	0 (0)	0 (0)	NA
NEUROPSYCHIATRIC					
Cognitive impairent, %	0 (0)	0 (0)	0 (0)	0 (0)	NA
Seizures requiring therapy for 6 months, %	0 (0)	0 (0)	0 (0)	0 (0)	NA
Cerebral vascular accident ever, %	2 (0.8)	1 (4.5)	0 (0.0)	1 (0.5)	0.136
Resection not for malignancy, %	0 (0)	0 (0)	0 (0)	0 (0)	NA
Cranial or peripheral neuropathy (excluding optic), %	3 (1.3)	0 (0.0)	1 (7.1)	2 (1.0)	0.120
Transverse myelitis, %	0 (0)	0 (0)	0 (0)	0 (0)	NA
RENAL					
Estimated or measured GFR <50%, %	0 (0)	0 (0)	0 (0)	0 (0)	NA
Proteinuria 24 h, ≥ 3.5 g, %	2 (0.8)	1 (4.5)	1 (7.1)	0 (0.0)	0.003
End-stage renal disease (regardless of dialysis or transplantation)	0 (0)	0 (0)	0 (0)	0 (0)	NA
Pulmonary hypertension, %	236 (100.0)	22 (100.0)	14 (100.0)	200 (100.0)	NA
Pulmonary fbrosis, %	6 (2.5)	0 (0.0)	0 (0.0)	6 (3.0)	0.575
Shrinking lung, %	0 (0)	0 (0)	0 (0)	0 (0)	NA
Pleural fibrosis, %	0 (0)	0 (0)	0 (0)	0 (0)	NA
Pulmonary infarction OR resection not for malignancy, %	0 (0)	0 (0)	0 (0)	0 (0)	NA
CARDIOVASCULAR					
Angina OR caronary artery bypass, %	0 (0)	0 (0)	0 (0)	0 (0)	NA
Myocardial infarction ever, %	0 (0)	0 (0)	0 (0)	0 (0)	NA
Cardiomyopathy, %	1 (0.4)	0 (0.0)	0 (0.0)	1 (0.5)	0.914
Vascular disease, %	0 (0)	0 (0)	0 (0)	0 (0)	NA
Pericarditis x 6 months or pericardiectomy, %	0 (0)	0 (0)	0 (0)	0 (0)	NA
PERIPHERAL VASCULAR					
Claudicatlon x 6 months, %	0 (0)	0 (0)	0 (0)	0 (0)	NA
Minor tissue loss (pulp space), %	0 (0)	0 (0)	0 (0)	0 (0)	NA
Significant tissue loss ever, %	0 (0)	0 (0)	0 (0)	0 (0)	NA
Venous thrombosis with swelling, ulceration, OR venous stasis, %	0 (0)	0 (0)	0 (0)	0 (0)	NA
GASTROINTESTINAL					
Infarction or resection of bowel (below duodenum), spleen, liver orgall bladder ever, %	1 (0.4)	0 (0.0)	0 (0.0)	1 (0.5)	0.914
Mesentaric insufhciency, %	0 (0)	0 (0)	0 (0)	0 (0)	NA
Chronic peritonitis, %	0 (0)	0 (0)	0 (0)	0 (0)	NA
Stricture OR upper gastrointestinal tract surgery ever, %	0 (0)	0 (0)	0 (0)	0 (0)	NA
Pancreatic insuffency requiring enzyme replacement or with pseudocyst, %	0 (0)	0 (0)	0 (0)	0 (0)	NA
MUSCULOSKELETAL					
Atrophy or weakness, %	0 (0)	0 (0)	0 (0)	0 (0)	NA
Deforning or erosive arthritis, %	0 (0)	0 (0)	0 (0)	0 (0)	NA
Osteoporosis with fracture or vertebral collapse, %	0 (0)	0 (0)	0 (0)	0 (0)	NA
Avascular necrosis, %	13 (5.5)	1 (4.5)	1 (7.1)	11 (5.5)	0.946
Osteomyelitis, %	0 (0)	0 (0)	0 (0)	0 (0)	NA
Ruptured tendons, %	0 (0)	0 (0)	0 (0)	0 (0)	NA
SKIN					
Alopecia, %	0 (0)	0 (0)	0 (0)	0 (0)	NA
Extensive scarring or panniculurn other than scalp and pulp space, %	0 (0)	0 (0)	0 (0)	0 (0)	NA
Skin uiceration (excluding thrombosis) for more than 6 months, %	0 (0)	0 (0)	0 (0)	0 (0)	NA
PREMATURE GONADAL FAILURE	18 (7.6)	2 (9.1)	0 (0.0)	16 (8.0)	0.532
DIABETES (regardless of treatment)	0 (0)	0 (0)	0 (0)	0 (0)	NA
MALIGNANCY(Exclude dysplasia)	1 (0.4)	0 (0.0)	0 (0.0)	1 (0.5)	0.914
